# Soluble ***α***-Klotho Serum Levels in Chronic Kidney Disease

**DOI:** 10.1155/2015/872193

**Published:** 2015-03-19

**Authors:** Silverio Rotondi, Marzia Pasquali, Lida Tartaglione, Maria Luisa Muci, Giusy Mandanici, Cristiana Leonangeli, Silvia Sales, Alessio Farcomeni, Sandro Mazzaferro

**Affiliations:** ^1^Department of Cardiovascular, Respiratory, Nephrology, Geriatric, and Anesthetic Sciences, “Sapienza” University, 5 Piazzale Aldo Moro, 00185 Rome, Italy; ^2^Department of Public Health and Infectious Diseases, Section of Statistics, “Sapienza” University, 5 Piazzale Aldo Moro, 00185 Rome, Italy

## Abstract

Transmembrane *α*-Klotho (TM-Klotho), expressed in renal tubules, is a cofactor for FGF23-receptor. Circulating soluble-*α*-Klotho (s-Klotho) results from TM-Klotho shedding and acts on Phosphate (P) and Calcium (Ca) tubular transport. Decreased TM-Klotho, described in experimental chronic kidney disease (CKD), prevents actions of FGF23 and lessens circulating s-Klotho. Thus, levels of s-Klotho could represent a marker of CKD-MBD. To evaluate the clinical significance of s-Klotho in CKD we assayed serum s-Klotho and serum FGF23 in 68 patients (age 58 ± 15; eGFR 45 ± 21 mL/min). s-Klotho was lower than normal (519 ± 183 versus 845 ± 330 pg/mL, *P* < .0001) in renal patients and its reduction was detectable since CKD stage 2 (*P* < .01). s-Klotho correlated positively with eGFR and serum calcium (Cas) and negatively with serum phosphate (Ps), PTH and FGF23. FGF23 was higher than normal (73 ± 51 versus 36 ± 11, *P* < .0002) with significantly increased levels since CKD stage 2 (*P* < .001). Our data indicate a negative effect of renal disease on circulating s-Klotho starting very early in CKD. Assuming that s-Klotho mirrors TM-Klotho synthesis, low circulating s-Klotho seems to reflect the ensuing of tubular resistance to FGF23, which, accordingly, is increased. We endorse s-Klotho as an early marker of CKD-MBD.

## 1. Introduction

Alpha-Klotho (Klotho) is a transmembrane (TM) protein primarily expressed in the kidney distal tubular cells [1] where it acts as an obligate coreceptor for the bone derived protein fibroblast growth factor-23 (FGF23) [2, 3]. In fact, TM-Klotho is required for FGF23 regulation of both renal handling of phosphate and renal synthesis of calcitriol [4]. At variance with TM-Klotho, soluble *α*-Klotho (s-Klotho) is the circulating protein resulting from the shedding of the extracellular domain of TM-Klotho operated by two metalloproteinases of the ADAM (a disintegrin and metalloproteinase domain-containing protein) family: ADAM 10 and ADAM 17 [5]. Importantly, s-Klotho also acts as a paracrine substance (with no receptor identified so far) with specific and FGF23 independent renal and extra-renal effects [6, 7]. In particular, s-Klotho inhibits the sodium-phosphate cotransporter NaPi2a expression in the proximal tubules thus generating a phosphaturic effect additive to and independent of FGF23 [7, 8] and activates the ion channel TRPV5 in the distal tubules, thus increasing tubular reabsorption of calcium [9]. In summary, *α*-Klotho, with its transmembrane and soluble forms, is deeply involved with the physiologic regulation of mineral metabolism [10].

Experimental models of CKD evidence early reduction of renal Klotho mRNA expression and of both TM-Klotho and s-Klotho [11, 12], and this is considered responsible for the development of kidney tubular cell resistance to FGF23. In agreement, in a model of selective Klotho deletion in the distal tubule, bone synthesis of FGF23 increased possibly aimed at overcoming tubular resistance; as a result fractional excretion of phosphate increased and calcitriol synthesis declined. These animal models have been invoked to describe the early changes that in renal patients anticipate the development of the newly identified CKD-MBD syndrome [13] or, as pointed out in a recent paper, disorder [14].

Available data on serum levels of s-Klotho in CRF patients almost invariably describe reduced values, a finding referred to a primary proportional reduction of TM-Klotho, as described in animal studies [16–18]. Moreover, circulating serum levels of s-Klotho are not always correlated with estimated glomerular filtration rate (eGFR) [19–22]. Excessive variability of some of the commercial kits available for s-Klotho assay in humans, as described by Heijboer et al. [23] could contribute, at least partially, to these conflicting results. Actually, Klotho assays have not been largely validated and various forms of circulating alpha-klotho could be responsible for this variability. Nonetheless, a very recent paper based on renal biopsies of patients with glomerulonephritis and variable renal damage, for the first time showed reduced tubular TM-Klotho expression associated with reduced serum levels of s-Klotho. Importantly, these patients had almost undetectable changes in the standard markers of mineral metabolism [24]. As a whole, the diagnostic role of the assay of s-Klotho as a useful biomarker of early mineral metabolism derangements in CKD patients may be relevant but warrants further research [19–22].

In the present study, we evaluated the diagnostic performance of s-Klotho in our CKD population of patients by using the most accredited commercial s-Klotho assay [23]. We aimed at confirming the reduction of circulating levels of s-Klotho and at verifying the links with FGF23 and with other markers of mineral metabolism derangements.

## 2. Methods

### 2.1. Subjects

We enrolled in the study, from our outpatient unit, eligible patients who gave informed consent. Inclusion criteria were white race, age 18–80 years, on conservative therapy with no evidence of acute underlying illness and naïve to treatment with any active or precursor metabolite of vitamin D.

Fasting blood samples were drawn from all participants to measure creatinine (Crs), albumin, calcium (Ca), phosphate (P), parathyroid hormone (PTH), 25(OH)-vitamin D (25D), 1,25(OH)_2_-vitaminD (1,25D), fibroblast growth factor-23 (FGF23), and soluble-*α*-Klotho (s-Klotho). We also collected fasting spot urine samples from all participants at the time of blood sampling to measure creatinine, phosphate, and calcium. In each patient, we recorded clinical parameters and prescribed therapies.

We classified CKD stage according to the National Kidney Foundation Disease Outcomes Quality Initiative clinical practice guidelines (KDOQI) [15].

Thirty normal subjects, recruited among the employees and fellows attending our Unit, served as control to obtain our reference values in particular for s-Klotho and FGF23. They were 14 males and 16 females, 35.0 ± 12.4 years of age with normal renal function (eGFR: 105.8 ± 15.4 mL/min/1,73 m^2^) negative urinary dipstick and no evidence of acute or chronic underlying illness.

### 2.2. Assays

Serum creatinine (kinetic alkaline picrate method), albumin (bromcresol purple method), Ca (cresolphthalein-complexone method), and P (ammonium molybdate method) were assayed by routine, standard colorimetric techniques with a Technicon RA-500 analyzer (Bayer Corporation Inc, Tarrytown, NY).

Serum PTH was assayed by an immunoradiometric technique (DiaSorin, Stillwater, MN, USA) based on a double antibody against the intact molecule; our normal values are within 10–55 pg/mL, with intra- and interassay variations of 6.5% and 9.8%, respectively.

Serum 25D determination was done with a commercial kit (Dia-Sorin, Stillwater, MN, USA) that included sample purification with acetonitrile followed by a ^125^I-based radioimmunoassay. Intra- and interassay coefficients of variation were 10.8% and 9.4%, respectively.

Serum levels of 1,25D were measured with a radioimmunoassay according to the manufacturer's protocol (IDS Ltd, Boldon, UK) including a monoclonal immunoextraction, followed by quantitation with a standard ^125^I-based radioimmunoassay. Intra- and interassay coefficients of variation were <12% and <14%, respectively. The normal range observed in our laboratory was between 19.5 and 67.0 pg/mL.

Serum levels of FGF23 were assayed with a commercially available kit (Kainos Lab Inc., Tokyo, Japan) that utilizes a 2-site ELISA for the full-length molecule. Two specific murine monoclonal antibodies recognized the biologically active FGF23, with a lower limit of detection of 3 pg/mL, and inter- and intraassay coefficients of variation of <5%. This assay has been demonstrated to be the most sensitive among the 3 different methods available [25]. In 30 normal subjects, we obtained a mean value of 29.8 ± 10.9 pg/mL (range 18–52 pg/mL), in line with reported data [25].

Serum levels of soluble alpha-Klotho were assayed with a novel enzyme-linked immunosorbent assay (ELISA) method detecting human s-Klotho developed first by establishing a monoclonal antibody with strong affinity for human Klotho protein, recognizing with high selectivity the tertiary protein structure of its extracellular domain (Immuno-Biological Laboratories Co., Ltd.). This ELISA system can specifically detect and measure the circulating serum s-Klotho levels in humans [26]. It was recently tested by Heijboer et al. [23] and the within- and between-run variation of the *α*-Klotho IBL was <5 and <8%, respectively. Measurements in serum and EDTA plasma samples were in agreement (*R* ^2^ = 0.99; *n* = 20) and linearity was tested by dilution in two samples with a concentration of 1929 and 2864 pg/mL. In one sample, 2-, 4-, and 8-time dilutions gave results as expected (100–117% of expected values). In our experience, in 30 normal subjects we obtained a mean value of 845 ± 330 pg/mL (range 2048–481 pg/mL).

We estimated glomerular filtration rate (eGFR) according to the abbreviated modification of diet in renal disease (MDRD) equation (eGFR = 186  ×  serum creatinine^−1.154^  ×  age^−0.203^  ×  0.742 if female) [27].

### 2.3. Statistical Analysis

Data are expressed as means ± SD for Gaussian variables or median and IQR when normality was not tenable.

We used Kolmogorov-Smirnov test to evaluate normality of continuous measurements.

Spearman correlation was used to assess monotonic covariation of measurements. Tests on Spearman correlation were Bonferroni adjusted for multiplicity. Nonparametric ANOVA (Kruskall-Wallis test) was used to compare measurements among groups and post-hoc comparisons in pairs were conducted by Bonferroni adjusted Mann-Whitney tests.

Log-measurements were confirmed to be normally distributed and were used as outcomes in multivariate regression models. The final multivariate model was obtained by minimizing the Akaike information criterion via a forward stepwise regression. All tests are two tailed and (adjusted) *P*-values <0.05 were considered as statistically significant.

Analyses were performed using the open source software package R version 3.0.2.

## 3. Results

For this study we recruited sixty-eight CKD patients, and their clinical and biochemical parameters are reported in Tables 1 and 2. Renal function, as reflected by eGFR, averaged 45 ± 21 mL/min (median ± SD) and included CKD stages from 2 to 4 (stage 2, *n* = 22 (32%); stage 3, *n* = 28 (41%); stage 4, *n* = 18 (27%)).

### 3.1. s-Klotho, FGF23, and eGFR

s-Klotho levels in our CKD patients were significantly lower (519 ± 183 versus 845 ± 330 pg/mL, *P* < .0001, Table 3) and FGF23 levels were significantly higher (73 ± 51 versus 36 ± 11 pg/mL, *P* < 0002, Table 3) than our reference values.

**Figure 1:**
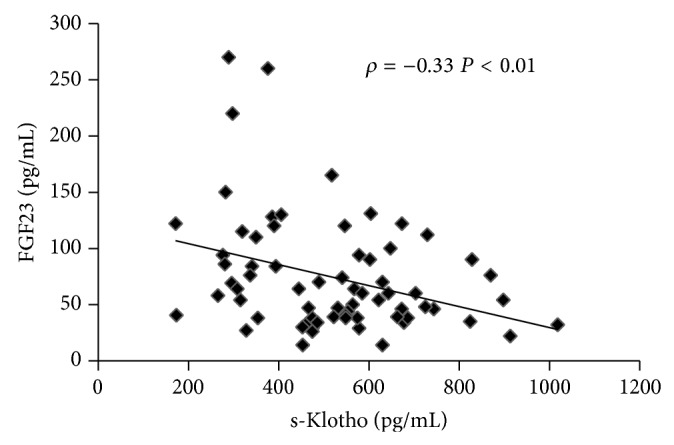
Correlation test of s-Klotho levels with FGF23 levels in CKD.

**Figure 2:**
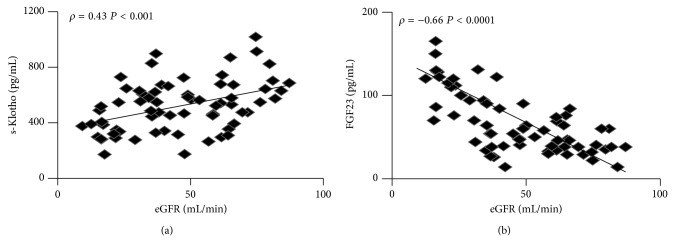
Correlation tests of eGFR with s-Klotho levels (a) and FGF23 (b).

In patients s-Klotho and FGF23 were negatively correlated (*ρ* = −0.33,  *P* < .01, Figure 1). In addition, there was a positive correlation between eGFR and s-Klotho (*ρ* = 0.43,  *P* < .001, Figure 2(a)) and a negative correlation between eGFR and FGF23 (*ρ* = −0.66,  *P* < .0001, Figure 2(b)).

**Figure 3:**
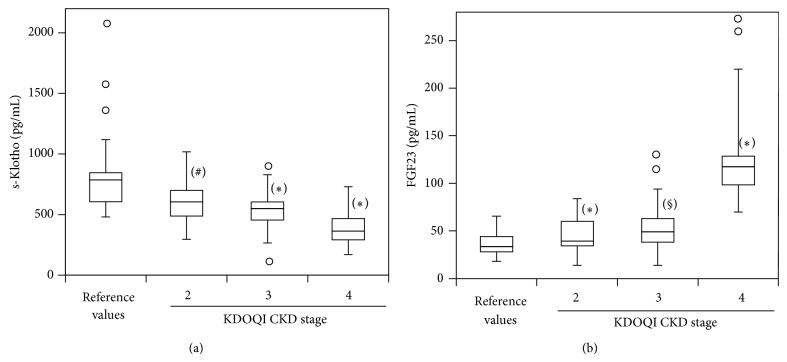
s-Klotho (soluble Klotho) (a) and FGF23 (fibroblast growth factor 23) and (b) serum levels stratified by CKD stage and compared with reference values. Indicated are medians, first and third quartiles, minimal and maximal values. Values between 1.5 and 3 times IQR above the third quartile or below the first are represented by circles. Wilcoxon rank-sum tests. KDOQI: National Kidney Foundation Disease Outcomes Quality Initiative clinical practice guidelines [15]. ^#^ *P* < .01 versus reference value; ^*^ *P* < .001 versus reference value; ^§^ *P* < .002 versus reference value.

When we evaluated s-Klotho in the different CKD stages, we found reduced levels since CKD stage 2, with a more significant reduction in CKD stages 3 and 4 (reference values = 845 ± 330, pg/mL; stage 2 = 611 ± 191, pg/mL, *P* < .01; stage 3 = 529 ± 160, pg/mL, *P* < .001; stage 4 = 393 ± 142, pg/mL; *P* < .001.  Figure 3(a)). Levels of FGF23 were significantly higher since CKD stage 2 (reference values = 36 ± 11, pg/mL; stage 2 = 46 ± 18, pg/mL, *P* < .001; stage 3 = 57 ± 28, pg/mL, *P* < .002; stage 4 = 136 ± 58, pg/mL, *P* < .001.  Figure 3(b)) with no further increment in later CKD stage. Multivariate analysis performed to identify the best predictors of s-Klotho (Table 4) and FGF23 (Table 5) included eGFR and serum calcium for s-Klotho and eGFR and 1,25D serum level for FGF23.

### 3.2. s-Klotho, FGF23, and Mineral Metabolism

**Figure 4:**
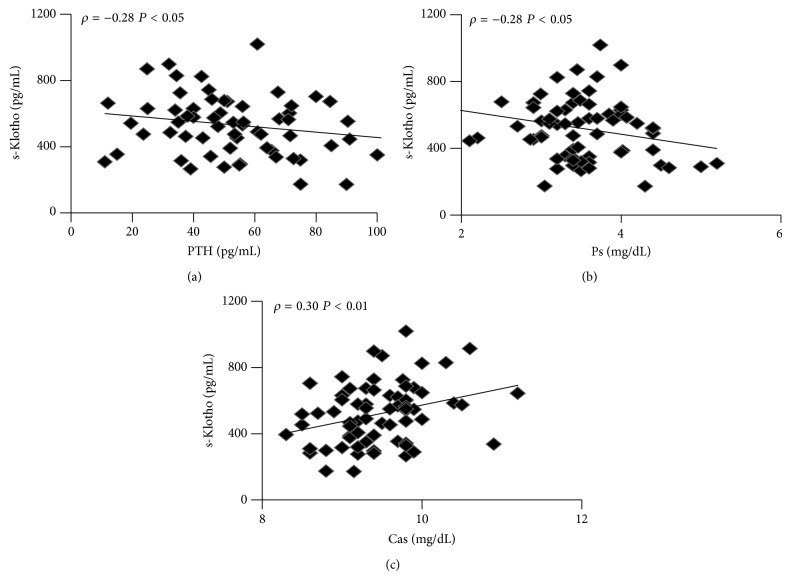
Correlation tests of s-Klotho levels with: (a) PTH, (b) Ps, (c) Cas.

s-Klotho correlated negatively with PTH (*ρ* = −0.28,  *P* < .05, Figure 4(a)) and Ps (*ρ* = −0.28,  *P* < .05, Figure 4(b)) and positively with Ca (*ρ* = 0.30,  *P* < .01, Figure 4(c)) while no correlation was found between s-Klotho and 1,25D, FE_PO_4__, and FE_Ca_.

**Figure 5:**
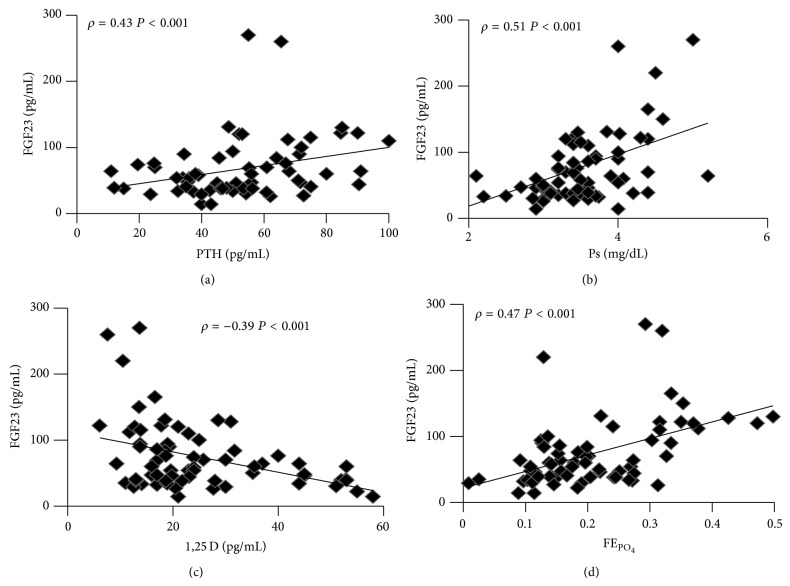
Correlation tests of FGF23 with: (a) PTH, (b) Ps, (c) 1,25D, (d) FE_PO_4__.

FGF23 levels correlated positively with PTH (*ρ* = 0.43,  *P* < .001, Figure 5(a)), Ps (*ρ* = 0.51,  *P* < .001, Figure 5(b)) and FE_PO_4__ (*ρ* = 0.47,  *P* < .001, Figure 5(d)) and negatively with 1,25D (*ρ* = −0.39,  *P* < .001, Figure 5(c)). No correlation existed with Ca and FE_Ca_.

## 4. Discussion

In agreement with experimental models of CKD [11, 12] and with papers in the literature [19, 21] our patients showed markedly reduced s-Klotho serum levels as compared to reference control. Moreover, our mean values in CKD and in normal controls are comparable to those available in papers the literature that employ our same method of assay [19, 28–30]. It is therefore suggested that although s-Klotho proteins are not homogeneous, consistent data can be obtained by different groups.

Renal function negatively affected s-Klotho levels with detectable reduction starting from CKD stage 2. Since s-Klotho is normally excreted in the urine [7–9], this reduction in serum levels along with progressive renal damage can be most probably explained by reduced renal synthesis. In fact, in case of stable renal Klotho production, a reduced excretion due to renal damage would increase serum levels. Alternatively, if renal excretion persisted to be normal, serum levels would not predictably decrease. On the contrary, an increased renal excretion seems highly improbable since this would contradict the available clinical [31] and experimental [32] data of reduced urinary s-Klotho in CKD. In our experience, the best predictor of s-Klotho was eGFR and this is confirmatory of the hypothesis we have just considered. The other positive predictor of s-Klotho in our study was serum Ca as if a regulatory role of s-Klotho would be present even in case of renal failure. This seems possible if we recall the direct effect of s-Klotho on TRPV5. However, the pathomechanism may not be straightforward since we did not find correlation between s-Klotho and FE_Ca_. Therefore, we should guess a more complex interplay with other changing parameters like eGFR, PTH, and 1,25D.

The negative relationship we found between s-Klotho and serum P is interesting due to the reported direct effect of circulating s-Klotho on the renal expression of NaPi2a. However, no correlation was evident with renal FE_PO_4__. Since s-Klotho and FGF23 correlate negatively in CKD but exert a similar positive effect on renal expression of NaPi2a, it is possible that the action of s-Klotho is shadowed by FGF23. Finally, the negative relationship with PTH should be regarded as secondary to reduced renal function.

Serum levels of FGF23 were higher than normal in our CKD population with increments detectable since CKD stage 2. Correlations of FGF23 were negative with eGFR and 1,25D, and positive with PTH, Ps and FE_PO_4__. Multivariate analysis evidenced eGFR and 1,25D as best predictors. These data suggest that bone cells somehow sense the reduction of eGFR very early and increase FGF23 synthesis mainly aiming at reducing 1,25D synthesis. In agreement, in a mice model of CKD administration of FGF23 antibodies resulted in a significant and dose dependent increase of 1,25D [33]. In our CKD population, the consensual increments of serum P and PTH along with that of FGF23 can be regarded, at variance, as mainly secondary to the reduction of renal function.

Interestingly in our study, a negative relationship emerged between s-Klotho and FGF23. This favors the hypothesis that s-Klotho, whose circulating levels are strongly related to renal function and are most probably secondary to reduced expression of TM-Klotho, can be regarded as a sensitive biomarker of TM-Klotho expression, useful to appreciate early development of tubular resistance to FGF23. Consequently, bone synthesis of FGF23 is increased. A recent paper with histologic data from patients with glomerulonephritis showed parallel reduction of renal Klotho and of s-Klotho together with increments in FGF23, which is in agreement with our data [24]. Transgenic animals selectively null for Klotho throughout the nephron, as reported recently by Lindberg et al., have negligible shedding of Klotho from renal explants and circulating levels reduced by 80%, thus revealing the kidney as a major contributor of circulating Klotho [34].

Significantly in our study, levels of s-Klotho and of FGF23 are roughly halved and, respectively, doubled in a population with an average eGFR of 45 mL/min/1,73 but with substantially normal values of Ca and P and with mean values of 1,25D and of PTH, respectively, at the lower and higher upper limits of normality. This confirms that changes in serum Ca and P are not reliable to detect early CKD-MBD, while s-Klotho seems more sensitive than PTH and 1,25D.

Limitations of our study are several. First, the number of evaluated patients is rather low. In fact, an increased number of cases would have reinforced the reliability of our results that, however, are in line with published data in the literature. Second, we did not include CKD stage 1 patients and this does not allow us to verify if s-Klotho diminish even in case of “normal” renal function. Certainly, this important issue warrants further investigations. Third, we did not measure urinary s-Klotho. Effectively, evidence of reduced urinary Klotho in our experience would have reinforced the hypothesis of reduced renal synthesis. However, scanty, available papers report reduced urinary Klotho in patients with renal failure [31] and in animals selectively null for nephron Klotho [34], both results confirming our hypothesis.

In conclusion, our results favor the hypothesis that renal Klotho synthesis diminishes early in renal disease and that s-Klotho proportionally lessens; bone detects these changes somehow and increases FGF23 production. Accordingly, s-Klotho represents an early marker of renal damage and of ensuing CKD-MBD, indicative of the cross-talk between bone and kidney.

## Figures and Tables

**Table 1:** Clinical characteristics of the CKD patients.

	CKD
Number of patients	68
M/F	37/31
Mean age, years	58 ± 15
eGFR, mL/min	45 ± 21
Mean body mass index	27 ± 4
Diabetes, number (%)	7 (10%)
Renal diagnosis	
Glomerulonephritis/vasculitis	16 (24%)
Interstitial nephritis	15 (22%)
Hypertensive/large vessel disease	18 (26%)
Hereditary nephropathy	4 (6%)
Unknown or missing data	15 (22%)
Therapies	
ACE inhibitor	28 (41%)
Angiotensin II receptor blockade	13 (19%)
Beta blocker	15 (22%)
Diuretic	10 (15%)
Insulin or oral hypoglycemic agent	6 (9%)

Values are mean ± standard deviation.

M/F: men/female; eGFR estimated glomerular filtration rate; ACE: angiotensin converting enzyme.

**Table 2:** Biochemistries in CKD.

Number of patients	68
Cas mg/dL	9,4 ± ,6
Ps mg/dL	3,5 ± ,7
PTH pg/mL	60,0 ± 31,7
25D ng/mL	23,7 ± 11,1
1,25D pg/mL	24,8 ± 13,2

Values are mean ± standard deviation.

Cas: serum calcium; Ps: serum phosphate; PTH: parathyroid hormone; 25D: 25(OH)-vitaminD; 1,25D: 1,25(OH)_2_-vitaminD.

**Table 3:** s-Klotho and FGF23 in CKD versus reference value.

	CKD (68)	Reference values (30)	*P*=
s-Klotho, pg/mL	519 ± 183	845 ± 330	.0001
FGF23, pg/mL	73 ± 51	36 ± 11	.0002

Mann-Whitney test; values are mean ± standard deviation.

s-Klotho: soluble-Klotho; FGF23: fibroblast growth factor-23.

**Table 4:** Multivariate analysis performed in CKD to identify predictors of s-Klotho.

VAR	Coef.	CI	*P*=
eGFR, mL/min	.006	.002, .010	.004
Cas, mg/dL	.193	.034, .035	.020

VAR: variable; coef: coefficient of linear regression; CI: confidence interval.

**Table 5:** Multivariate analysis performed in CKD to identify predictors of FGF23.

VAR	Coef.	CI	*P*=
eGFR, mL/min	−.019	−.023, −.014	.0001
1,25D, pg/mL	−.016	−.024, −.008	.0001

VAR: variable; coef.: coefficient of linear regression; CI: confidence interval.
